# Experimental Study on the Mechanical Properties, Water Absorption, and Fiber Degradation of Naturally Aged Glass Fiber and Polypropylene Fiber-Reinforced Concrete

**DOI:** 10.3390/ma15113760

**Published:** 2022-05-24

**Authors:** Zhu Yuan, Yanmin Jia

**Affiliations:** School of Civil Engineering, Northeast Forestry University, Harbin 150040, China; zhu_yuan163@163.com

**Keywords:** fiber-reinforced concrete, naturally aged concrete, glass fiber, polypropylene fiber, water absorption, fiber-matrix interaction, fiber degradation

## Abstract

The main objective of this study is to better understand the performance changes of naturally aged glass fiber-reinforced concrete (GFRC) and polypropylene fiber-reinforced concrete (PPFRC), especially the degradation of fibers, which is of great significance for evaluating the durability of structures using these two types of composite materials. The mechanical properties, water absorption, and microstructures of GFRC and PPFRC at a curing age of three years, including their compressive strength, full curves of water absorption, fiber-matrix interaction, and fiber degradation, were systematically studied, and the related properties were compared with those at the curing age of 28 days. The degradation of fibers after freeze-thaw cycles was also studied. The results revealed the following. The water/binder ratio (w/b) affects the rate of increase of the long-term compressive strength of naturally aged concrete. In general, the water absorption of fiber-reinforced concrete (FRC) at the curing age of three years was found to be significantly reduced, but with the increases of w/b and the fiber content to the maximum values, the water absorption of the specimens cured for three years was higher than that of the specimens cured for 28 days. Moreover, with the increase of the curing age, the optimal glass fiber (GF) contents for reducing the water absorption decreased from 1.35% to 0.90% (w/b = 0.30), and from 0.90% to 0.45% (w/b = 0.35), respectively. The GF surface was degraded into continuous pits with diameters of about 200 to 600 nm, and the surface of the pits was attached with spherical granular C-S-H gel products with diameters of about 30 to 44 nm. The freeze-thaw cycles were found to have no significant effect on the pits on the GF surface and the granular C-S-H gel products attached to the pits, but caused a portion of the cement matrix covering the GF to fall off. The interfacial bonding between the polypropylene fiber (PPF) and the cement matrix exhibited almost no change in the PPFRC after three years of curing as compared with that after 28 days of curing. Furthermore, the cement hydration gel on the PPF surface was not significantly damaged by 150 freeze-thaw cycles.

## 1. Introduction

Concrete is the most commonly used building material worldwide, but in the service process of concrete structures, deterioration caused by physical and/or chemical factors often occurs; for example, chloride attack, sulfate attack, freeze-thawing, shrinkage, carbonization, alkali-silicon reaction, and steel corrosion, etc., seriously threaten the durability of concrete. Via in-depth research, it has been found that water is usually involved in various degradation processes. As a carrier of aggressive ion migration, water is a direct participant in physical degradation and an indirect participant in chemical degradation [1,2,3,4,5,6,7]. Concrete is a typical porous medium composed of a solid skeleton and pores filled with gas and water; these pores include capillary pores, pores, and micro-cracks. The durability of concrete in harsh environments depends largely on the impact of its pore system on the water transport performance [3,5,8,9]. As one of the three main mechanisms of water transport [5,6], water absorption is an effective method by which to characterize the ability of porous materials to absorb and transfer water through pores [2], and is an important factor for the quantification of the durability of cement systems [2,5,6]. The water absorption of concrete reflects its porosity and the volume of permeable pores and the connection between these pores [7]. As an important parameter with which to describe the durability of concrete, water absorption has been used in increasingly more standards and research. Improving the pore structure and connectivity to reduce water absorption and improve waterproof performance is an effective way to improve the durability of concrete [1,2,3,4,5,6,7,8,9,10,11,12].

Glass fiber (GF) and polypropylene fiber (PPF) are two excellent materials for the micro-reinforcement of concrete. GF is characterized by a low specific gravity, low water absorption, a high elastic modulus, and high tensile strength, thereby providing great potential for concrete reinforcement. PPF has attracted the attention of researchers due to its low cost, excellent toughness, and high resistance to shrinkage and cracking. The addition of short-cut GF or PPF forms a three-dimensional staggered support network inside concrete, thereby compacting its internal structure [1,13,14] and effectively improving its mechanical properties [14,15,16]. Moreover, GF and PPF are also used to improve the water absorption properties of concrete [1,14,15,16,17,18,19,20,21]. Studies have shown that the addition of GF and PPF to the concrete mixture will decrease pore size and porosity, an enhanced bond between C-S-H and Ca(OH)_2_, and a denser interfacial transition zone [1,13]. Some studies also suggest that the addition of GF can reduce the water absorption of concrete [14,15,16,17], while others indicate that it increases the water absorption rate [21].

Although GF and PPF have excellent engineering properties, the durability of fiber-reinforced concrete (FRC), especially the degradation of GF in concrete, limits its engineering applications to a certain extent [22,23,24,25,26,27]. Research has shown that the calcium hydroxide produced during cement hydration and solidification maintains the high pH value (approximately 12.6) of the interstitial solution in the pores of the cement matrix. This alkaline environment will cause the rapid degradation of GF, including via strength and weight loss and diameter reduction [22]. Degradation causes GF to change from a ductile to a brittle material, thereby counteracting its positive effect [23]. The most successful way to prevent this adverse effect is to use mineral admixtures and alkali-resistant GF in the concrete mixture [24]; however, alkali-resistant GF treated with zirconia has exhibited the same degradation phenomenon [22,26]. The incorporation of fly ash, slag, and silica fume can reduce degradation to a certain extent [22].

It takes a long amount of time to study the natural degradation of GF in GF-reinforced concrete (GFRC) via natural curing. Due to the lack of long-term performance data, researchers often use accelerated aging tests in lab conditions that simulate aging to study the durability of GFRC composites [27,28,29,30,31,32,33,34]. While hot water immersion is usually used as an accelerated aging method [28,29,34], some studies have found that this method is not accurate [28,34]. In contrast, weather conditions can be simulated more accurately by mixing freeze-thaw cycles and wet-dry cycles; nevertheless, it is necessary to determine the correlation between real weather and the proposed new accelerated aging method [28]. Research has indicated that PPF is chemically inert and very stable in the alkaline environment of concrete, and that PPF reinforced concrete (PPFRC) is suitable for alkaline environments [1]. However, there have been fewer studies on PPF interface regions compared with GF.

Based on the current research situation, the following problems are identified. First, while the compressive strength is an important index for concrete structure design, most studies on the compressive strength of GFRC and PPFRC have been conducted after curing for 28, 56, or 90 days, while there have been few studies on the long-term (more than 1 year) compressive strength. More importantly, the influences of the water/binder ratio (w/b) and fiber content on the development of the FRC strength with the increase of age have not been considered. Second, most studies on the water absorption of FRC only tested the water absorption at curing ages between 28 and 90 days [7,14,18,21], and did not conduct more long-term investigations. Finally, there remains a discrepancy between accelerated lab conditions and natural aging regarding the study of fiber degradation. The study of the fiber degradation of naturally aged FRC can provide a comparative analysis basis for the improvement of accelerated aging methods.

Research on the property changes of concrete naturally aged for several years, especially investigations of fiber degradation, would contribute to the durability design of FRC structures. In the present work, the properties of GFRC and PPFRC naturally aged for three years at room temperature, including the compressive strength, full curves of water absorption, fiber-matrix interaction, micro-morphology of the interfacial transition zone, and fiber degradation, were systematically studied. The test results were compared with those of samples cured for 28 days. Moreover, a rapid freeze-thaw cycle test was conducted to study the fiber degradation of the FRC aged for 3 years under freeze-thaw cycles. These investigations provide references for the design and evaluation of the structural durability of GFRC and PPFRC.

## 2. Materials and Methods

### 2.1. Raw Materials

#### 2.1.1. Ordinary Portland Cement

The P.O 42.5 ordinary Portland cement was used in this study, and its physical and mechanical properties are shown in Table 1. The testing standards for the physical and mechanical properties of cement are as follows. The soundness and setting time test procedure was conducted according to the standard GB/T 1346-2011 [35]. The specific surface area test procedure was conducted according to the standard GB/T 8074-2008 [36]. The strength test procedure was conducted according to the standard GB/T GB/T 17671-1999 [37]. The chemical compositions of cement are shown in Table 2.

#### 2.1.2. Fly Ash

The fly ash is Class I fly ash produced locally in Harbin, China. The chemical compositions of fly ash are shown in Table 3.

#### 2.1.3. Silica Fume

The chemical compositions of silica fume are shown in Table 4.

#### 2.1.4. Slag

The strength grade of the slag is S95, and the density is 2.89 g/cm^3^.The chemical compositions of slag are shown in Table 5.

#### 2.1.5. Fibers

**Figure 1:**
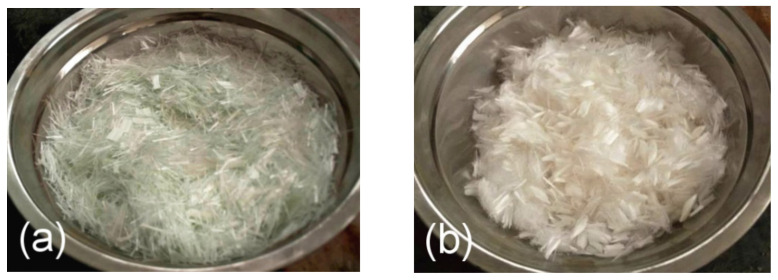
Appearance of the used fibers: (**a**) GF and (**b**) PPF.

The fibers used in the test are chopped GF and PPF with a length of 12 mm. The morphology and main parameters of the fibers used are shown in Figure 1 and Table 6.

#### 2.1.6. Aggregate

Natural river sand was selected as fine aggregate and its fineness modulus was Mx = 2.66. Coarse aggregate with 5–20 mm continually graded macadam and a 14.87% crushing value index was used.

#### 2.1.7. Water

The water used in the test is the municipal water supply for the city.

#### 2.1.8. Superplasticizer

A polycarboxylate-type superplasticizer was adopted to meet the requirement of a slump for fresh concrete.

### 2.2. Mix Proportions

The mixture designs were carried out according to the JGJ 55-2011 standard [38], and the mixture proportions of the concrete mixes are reported in Table 7.

### 2.3. Sample Preparation

The samples were prepared in accordance with the CECS13-2009 [39] and GB/T 50081-2002 [40] standards and were cubes of 100 × 100 × 100 mm^3^ in size. After 28 days of water curing under standard conditions, some of the samples were subjected to the corresponding performance test, and the others were cured at room temperature for three years. The room temperature was 24 ± 2 °C, and the humidity was 40% ± 3%.

### 2.4. Test Procedures

#### 2.4.1. Measurement of Compressive Strength

A total of 84 cubic samples were used for the compressive strength tests. According to the GB/T 50081-2002 standard [40], a YAW-2000A computer-controlled electro-hydraulic servo universal tester (made by Jinan Time Shijin Testing Machine Co., Ltd., Jinan, China) was used to test the compressive strength of the samples after curing for 28 days and three years, respectively. Three samples were tested in each group, and the average value of the test data was taken as the test result.

#### 2.4.2. Freeze-Thaw Cycle Test

**Figure 2:**
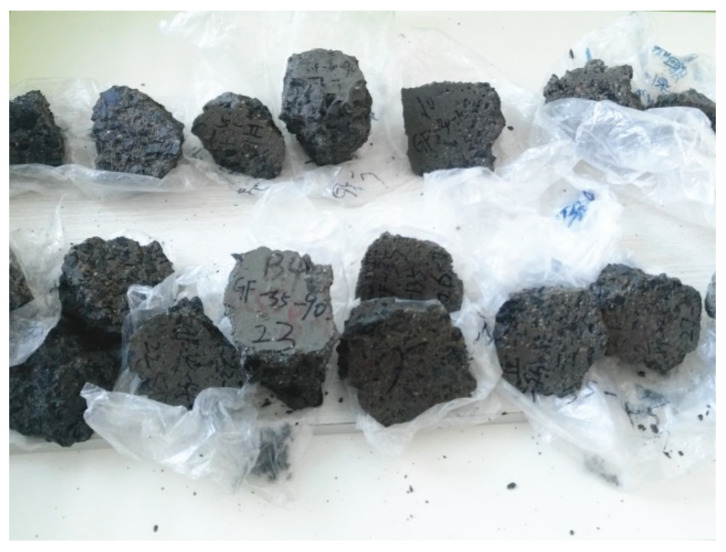
A portion of the blocks after freeze-thaw cycles.

The freeze-thaw cycle test was conducted according to the GB/T 50082-2009 standard [41]. A KDR-V5 concrete rapid freeze-thaw test chamber (made by Tianjin Shouke Testing Machine Co., Ltd., Tianjin, China) was used for the rapid freeze-thaw test. After completing the three-year compressive strength test, relatively large blocks (irregular polyhedrons with a diameter greater than 5 cm) were selected from the crushed samples. The debris on the surface of each block was gently wiped off manually, and the serial number was marked. The selected blocks were placed into the freeze-thaw test chamber for 150 freeze-thaw cycles. The upper limit of the temperature in the test chamber was 5 °C, and the lower limit was −16 °C. Each freeze-thaw cycle took 2.5 h. Because the surfaces of the blocks could be easily crushed, and slag could easily fall off, after the freeze-thaw cycles the samples were packed separately in plastic bags, as shown in Figure 2. These blocks were dried at room temperature for seven days and then subjected to SEM observation.

#### 2.4.3. Water Absorption Test

The 100 × 100 × 100-mm specimens were immersed in water for water absorption tests, and the test procedure was conducted according to the ASTM C642-2013 standard [42]. After the full curves of water absorption at the curing age of 28 days [14] were obtained, the samples were placed at room temperature to naturally age for three years. They were subsequently dried in an oven at 60 °C for 72 h, their mass was determined, and the full curves of water absorption at the curing age of three years were then obtained.

#### 2.4.4. SEM Observation

**Figure 3:**
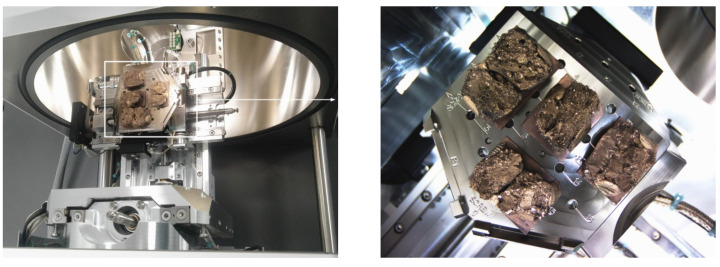
A small portion of the specimens placed onto the Apreo SEM scanning table.

The scanning electron microscopy (SEM) test procedure was conducted according to the ASTM C1723-2010 standard [43]. A Quanta 200 scanning electron microscope (made by FEI company, Eindhoven, The Netherlands) was used to observe the micromorphologies of the concrete specimens cured for 28 days, and an Apreo SEM (made by Thermo Fisher Scientific, Waltham, MA, USA) was used to observe the concrete specimens cured for three years. Figure 3 presents a small portion of the specimens with gold coating placed onto the Apreo SEM scanning table.

## 3. Results and Discussion

### 3.1. Compressive Strength

**Figure 4:**
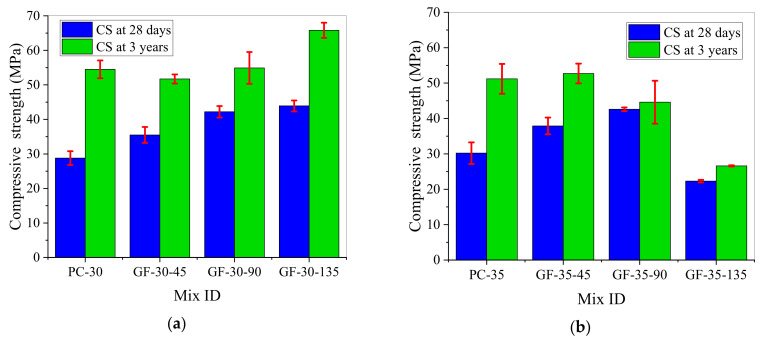
The 28-day and three-year compressive strengths of the GFRC: (**a**) w/b = 0.30; (**b**) w/b = 0.35.

Figure 4 exhibits the 28-day and three-year compressive strengths of the GFRC. According to Figure 4a, when w/b = 0.3, the 28-day compressive strength increased with the increase of the GF content, and reached the maximum value of 43.9 MPa when the GF content was 1.35%, thereby exhibiting an increase of 52.43% as compared with the value of 28.8 MPa achieved by specimen PC-30. The three-year compressive strength was found to generally increase with the increase of the fiber content, and reached the maximum value of 65.8 MPa when the GF content was 1.35%, but the rate of increase was only 20.73% as compared with the compressive strength of specimen PC-30 (54.5 MPa). Thus, it was determined that 1.35% was the optimal content of GF. This result is consistent with reference [18,44,45,46], that is, the addition of GF significantly increased the compressive strength. This is mainly due to the strong bond force between GF and the cement matrix, and the uniform distribution of GF in the cement matrix formed a three-dimensional network structure, which could inhibit the generation and propagation of cracks [14,47,48].

According to Figure 4b, when w/b = 0.35, the 28-day and 3-year compressive strengths of the GFRC first increased and then decreased with the increase of the GF content, and the optimal GF content decreased from 0.90% to 0.45% with the further increase of the curing age. Under the optimal fiber content, the 28-day compressive strength increased from 30.2 to 42.6 MPa, an increase of 41.1%, while the 3-year compressive strength increased from 51.2 to 52.7 MPa, an increase of only 2.9%.

**Figure 5:**
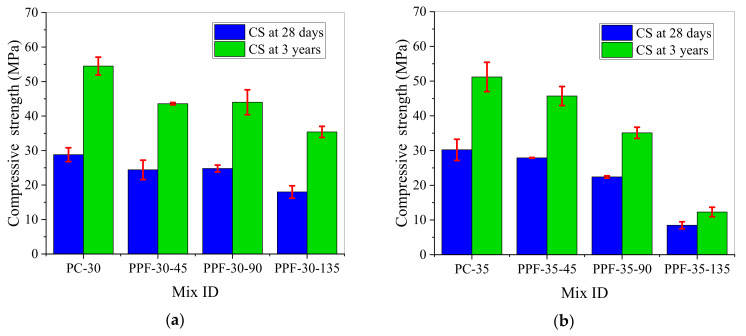
The 28-day and three-year compressive strengths of the PPFRC: (**a**) w/b = 0.30; (**b**) w/b = 0.35.

Figure 5 presents the 28-day and three-year compressive strengths of PPFRC. When w/b = 0.30 and 0.35, the compressive strength was reduced, and the 28-day and three-year compressive strengths both decreased with the increase of the PPF content. In reference [20,49], the addition of PPF also reduced the compressive strength of concrete, and the compressive strength decreased with increasing PPF dosage. Compared with those of specimens PC-30 and PC-35, the three-year compressive strengths of specimens PPF-30-135 and PPF-35-135 decreased from 54.5 and 51.2 MPa to 35.4 and 12.3 MPa, respectively, thereby exhibiting respective decreases of 35.0% and 76.0%.

In Table 8, I_3y-28d_ represents the rate of increase of the three-year compressive strength of concrete relative to the 28-day compressive strength. It is evident that when w/b = 0.30 and 0.35, the I_3y-28d_ value of the GFRC was significantly lower than those of specimens PC-30 and PC-35. At the same GF content, the I_3y-28d_ value of the GFRC with w/b = 0.30 was higher than that with w/b = 0.35. For the PPFRC, excluding the increase of the strength of specimen PPF-30-135 from 18 to 35.4 MPa (an increase of 96.67%), the other I_3y-28d_ values were lower than that of plain concrete, and the I_3y-28d_ values of the PPFRC were higher than those of the GFRC at the same fiber content. In other words, w/b and the fiber content affected the rate of increase of the long-term compressive strength of the naturally aged concrete; after w/b increased, as the contents of GF and PPF increased, the compressive strength of concrete increased more slowly.

### 3.2. Water Absorption

**Figure 6:**
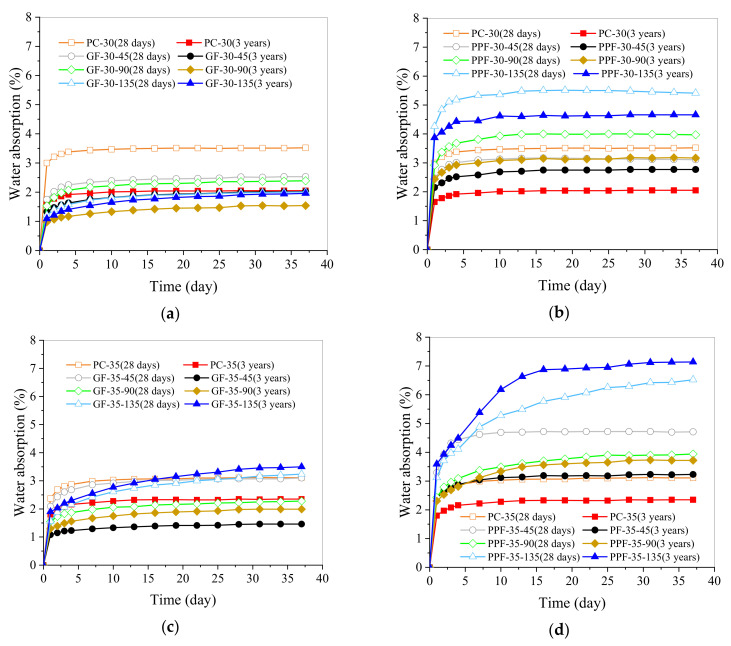
The full curves of the water absorption of the specimens: (**a**) Full curves of the water absorption of the GFRC when w/b = 0.30; (**b**) Full curves of the water absorption of the PPFRC when w/b = 0.30; (**c**) Full curves of the water absorption of the GFRC when w/b = 0.35; (**d**) Full curves of the water absorption of the PPFRC when w/b = 0.35.

Water absorption is a good index to measure the porosity of concrete. The durability of concrete can be estimated by porosity, because higher porosity will make it easier for harmful chemicals to penetrate into the concrete. These chemicals can react with its components and change the performance of concrete materials [9,15,21]. Table 9 exhibits the water absorption test results of the GFRC and PPFRC specimens cured for three years. The percentages in parentheses represent W_3y-28d_, which reflects the increase of water absorption compared to the specimens cured for 28 days [14]. When the value of W_3y-28d_ is negative, it indicates that the water absorption of the concrete aged for three years was lower than that of the concrete aged for 28 days; thus, the smaller the value of W_3y-28d_, the greater the rate of reduction of water absorption, and the more compact the inner structure of the concrete. Figure 6 presents the full curves of the water absorption of the GFRC and PPFRC at different curing ages. The water absorption data from the 31st day in the full curves were used as the measurement index.

It can be seen from Table 9 and Figure 6 that for the concrete specimens cured for three years, when w/b = 0.30, the addition of GF reduced the water absorption, and as the GF content increased, the water absorption first decreased and then increased. Specimen GF-30–90 had the lowest water absorption rate of 1.54%, which was 24.9% lower than that of specimen PC-30, namely 2.05%. Compared with specimen GF-30-90 cured for 28 days, the water absorption was reduced by 35%. If the water absorption of the specimens cured for 28 days is used to measure the effect of GF, 1.35% is the optimal GF content [14], and if the water absorption of the specimens cured for three years is considered, the optimum GF content is reduced to 0.90%.

When w/b = 0.30, after adding PPF, the water absorption of the concrete cured for three years increased, and the water absorption increased with the increase of the PPF content. The water absorption of specimen PPF-30-135 was 4.66%, which was 127.3% higher than that of specimen PC-30.

When w/b = 0.35, the water absorption of the GFRC increased with the increase of the GF content, and GF contents of 0.45% and 0.90% respectively reduced the water absorption by 37.6% and 15.0% as compared with that of specimen PC-35. In contrast, the GF content of 1.35% increased the water absorption by 47.9%. Thus, 0.45% is the optimal content of GF. In other words, with the increase of w/b to 0.35, when the effect of GF on the water absorption of the specimen aged for 28 days was considered, and 0.90% was found to be the optimal content [14]. However, when the curing age reached three years, the optimal content of GF decreased to 0.45%. In other words, with the increase of the curing age, the optimal GF content to reduce the water absorption of concrete decreased. Considering the long-term durability of concrete, the optimal GF content for concrete cured for 28 days (0.90%) may not be the best choice.

When w/b = 0.35, the influence of PPF on water absorption was the same as that when w/b = 0.30, and the water absorption increased with the increase of the PPF content. The water absorption of specimen PPF-35-135 increased from 2.34% (the water absorption of specimen PC-35) to 7.12%, thereby reflecting an increase of 204.3%.

According to the data presented in Table 9 and Figure 6, when w/b = 0.30, the W_3y-28d_ values of GFRC and PPFRC ranged from -3.0% to -35.0%, and the W_3y-28d_ value of PC-30 was −41.6%. This means that for the GFRC and PPFRC, the water absorption of the same samples cured for three years exhibited different degrees of reduction as compared to that of the samples cured for 28 days, but the water absorption of plain concrete had the highest rate of decrease. When w/b = 0.35, the W_3y-28d_ values of specimens GF-35-45 and PPF-35-45 were less than that of PC-35; the values were 9.1% and 10.9%, respectively, which means that when the fiber content increased to 1.35%, the water absorption rates of these two samples after curing for three years did not decrease, but increased. The reason for this is as follows. The water absorption test used the same sample at two different curing ages. During the first water absorption test and that after three years, the unreacted cementitious material inside the concrete continued to react with water to make the structure more compact, thereby reducing the water absorption. In reference [21], similar results were obtained. The water absorption of the fiber-reinforced concrete cured for 90 days was lower than that cured for 28 days. However, due to the large internal pore size and the number of pores in specimens GF-35-135 and PPF-35-135 [14], even if a portion of the unreacted cement continued to react, the resulting hydration products were not sufficient to effectively improve the pore distribution, resulting in an increase in water absorption at the curing age of three years.

**Figure 7:**
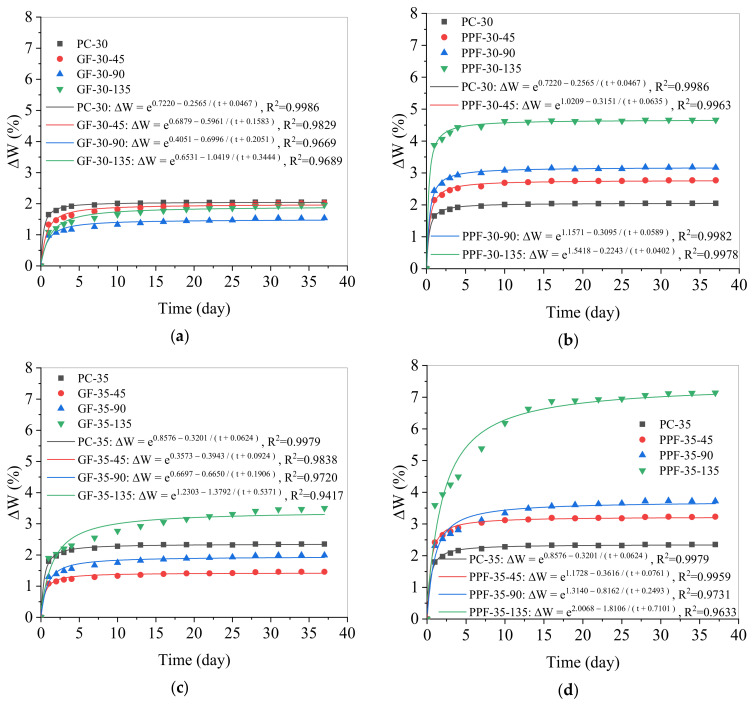
The fitting curves of the water absorption of concrete after curing for three years: (**a**) Fitting curve of the water absorption of the GFRC when w/b = 0.30; (**b**) Fitting curve of the water absorption of the PPFRC when w/b = 0.30; (**c**) Fitting curve of the water absorption of the GFRC when w/b = 0.35; (**d**) Fitting curve of the water absorption of the PPFRC when w/b = 0.35.

It can be seen from Figure 6 that the water absorption changed with time. In this study, the nonlinear relationship between water absorption and time is used to propose a new nonlinear formula that can describe the long-term water absorption process of concrete:

ΔW (t) = exp ((a + b)/(t + c))(1)

where a, b, and c are three fitting parameters, t represents time (days), and ΔW represents water absorption. Figure 7 presents the fitting curves of the long-term water absorption process and the water absorption formulas of plain concrete, GFRC, and PPFRC under different values of w/b. It can be seen from the figure that Equation (1) can well describe the change in water absorption of concrete at the curing age of three years (*R*^2^ is much larger than 0.9).

### 3.3. Microstructure

**Figure 8:**
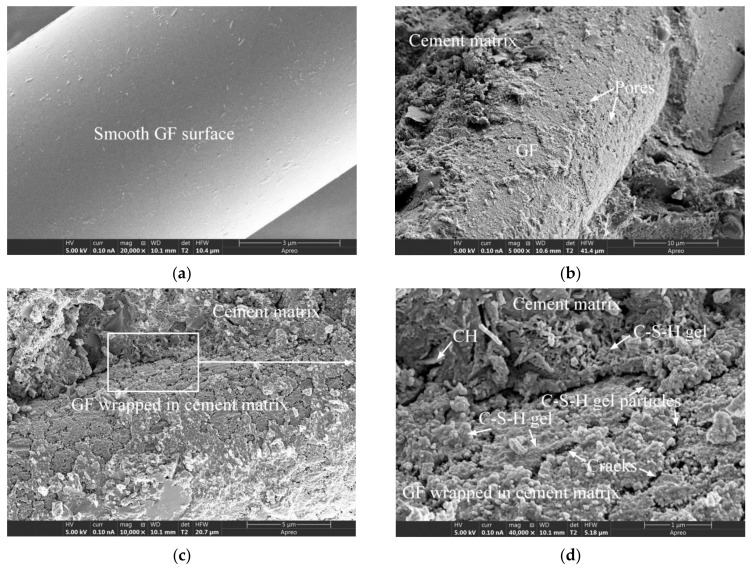
The SEM images of GF and GFRC specimens: (**a**) The unused GF; (**b**) GF-30-135; (**c**) GF-35-135; (**d**) GF-35-135.

**Figure 9:**
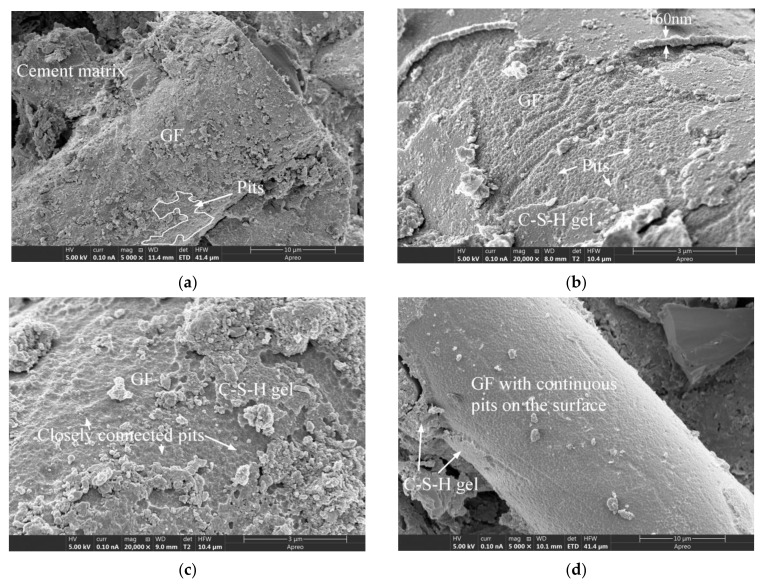
The SEM images of GFRC specimens: (**a**) GF-30-135; (**b**) GF-30-90; (**c**) GF-30-90; (**d**) GF-30-45.

**Figure 10:**
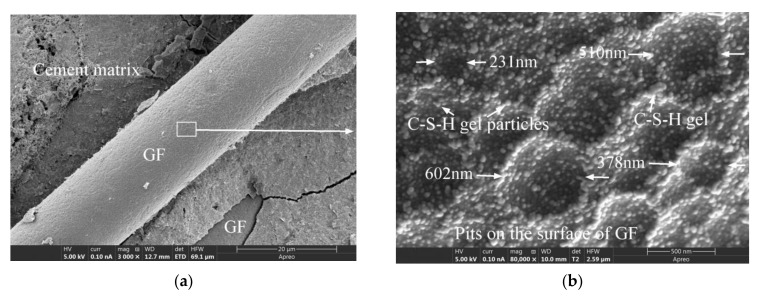

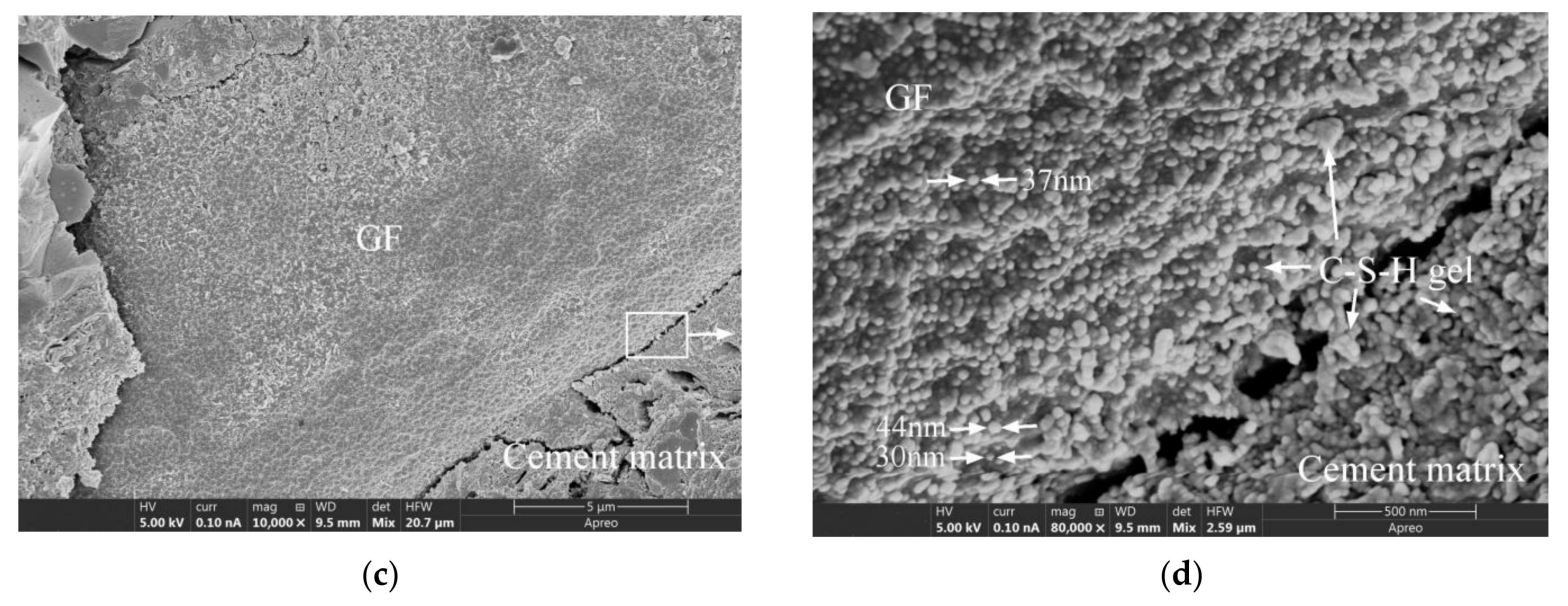
The SEM images of GFRC specimens: (**a**) GF-35-45; (**b**) GF-35-45; (**c**) GF-35-90; (**d**) GF-35-90.

Figure 8, Figure 9 and Figure 10 present the micromorphologies of the GFRC specimens cured for three years (freeze-thaw tests were not conducted). Figure 8a depicts unused GF that had been cleaned with anhydrous ethanol. Under 20,000× magnification, it is evident that the fiber surface was smooth and unetched. It can be seen from Figure 8b,c that the GF was well bonded to the cement matrix, and the cement matrix tightly covered the surface of the GF. Figure 8b reveals that there were many pores on the surface of the cement matrix. Figure 8d is a partial enlarged view of Figure 8c, from which it can be seen that the C-S-H gel wrapped with GF was flocculent and accompanied by a large number of cracks. As shown in Figure 8d, a large number of fine granular gel products were observed in the gap between the C-S-H flocculent gel. The outer cement matrix of the C-S-H flocculent gel encapsulating the GF was a hollow network of C-S-H gel with a small amount of calcium hydroxide (CH). The interface area had many pores and was not very compact, which may be a relatively weak link in GFRC. It may be difficult to completely eliminate these pores, but the following methods can be tried to reduce the size or quantity of these pores: The first is to adjust the content of mineral admixtures (fly ash, silica fume or slag) in the mixture proportions to make the concrete structure more compact and produce more crystals in the interface transition zone between the fiber and cement matrix to fill the pores [1,9]; The second is to add nano powder, such as nano silica, to reduce pores through the nano filling effect [9,18].

In specimen GF-30-135 shown in Figure 9a, it can be seen that the GF was well bonded to the cement matrix, and the surface of the GF was wrapped in the cement matrix. However, the position marked by curves in the figure did not cover the cement matrix, and there were many pits on the exposed GF surface. In specimen GF-30-90 depicted in Figure 9b,c, there were continuous and closely arranged pits on the surface of the GF where the C-S-H gel partially fell off. In the GF-30-45 specimen shown in Figure 9d, the C-S-H gel on a large portion of the surface of the GF fell off, and the exposed part was covered with the same pits as those shown in Figure 9a–c. This type of pit generally existed on the GF surface, which was caused by the degradation of the GF after being corroded by the cement hydration product. In a previous study [23], the degree of surface degradation of GF generated by accelerated aging was low and local, and the morphology of GF degradation was quite different from that caused by natural aging in the present investigation.

Figure 10a,c present the GF morphologies of specimens GF-35-45 and GF-35-90, it can be seen that after the cement hydrate gel product on the GF surface fell off, the exposed GF surface was also found to be covered with continuous pits, which is similar to Figure 9d. Figure 10b,d are partial enlarged views of Figure 10a,c, respectively. Figure 10b reveals that the diameters of the pits were about 200 to 600 nm, and the surface of the pits was scattered with C-S-H gel particles. Most of these particles were spherical or ellipsoidal in shape. As shown in Figure 10d, the gel product in the interface between the GF and the cement matrix and the gel product attached to the surface of the GF had highly consistent morphologies, both of which included a small amount of flocculent gel and a large number of spherical-like gel particles. The diameters of these spherical or ellipsoidal gel particles were approximately 30 to 44 nm. From the morphological perspective, the accumulation of a large number of spherical gel particles constituted the interfacial transition zone between the GF and the cement matrix. Although these intergranular pores in the interface region are considered to be too small to adversely affect the strength and permeability of the hydrated cement matrix [50], their presence may increase the weak links in the interface area and ultimately adversely affect the strength and durability of the concrete.

**Figure 11:**
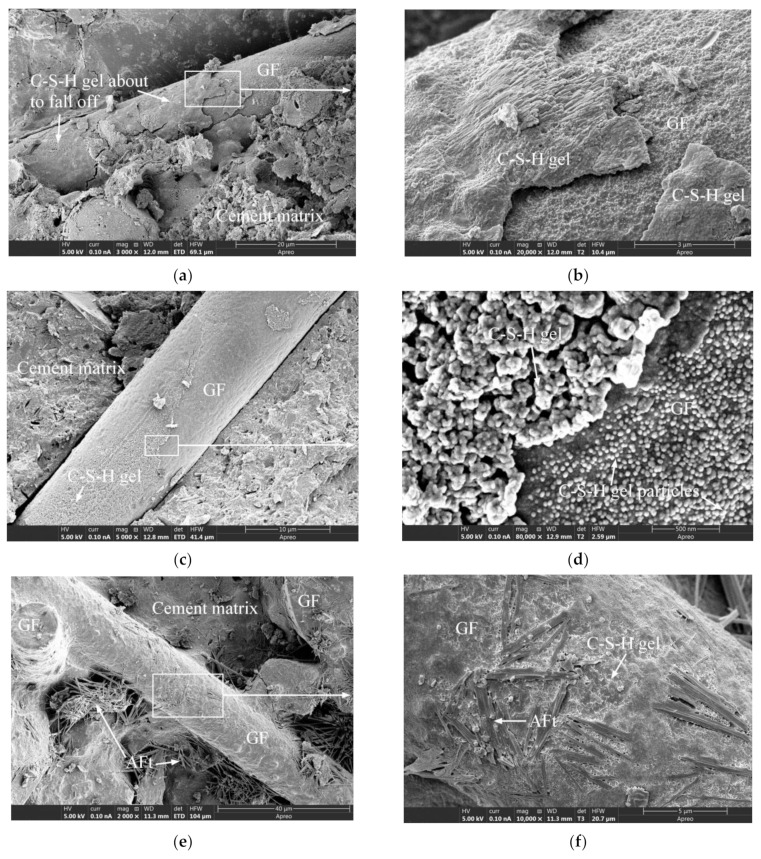
The SEM images of GFRC specimens after freeze-thaw cycles: (**a**) GF-30-135; (**b**) GF-30-135; (**c**) GF-30-90; (**d**) GF-30-90; (**e**) GF-30-135; (**f**) GF-30-135.

Figure 11 exhibits the microscopic morphologies of the GFRC after 150 freeze-thaw cycles. Figure 11b,d,f are local enlarged views of Figure 11a,c,e, respectively. It is evident that after 150 freeze-thaw cycles, the morphologies of the C-S-H gel on the GF surface can be categorized as one of the following three types: completely falling off, partially falling off, and not falling off.

It can be seen from Figure 11a,b that the C-S-H gel wrapped on the GF surface was about to fall off due to the effect of the freeze-thaw cycles. As shown in Figure 11c,d, the C-S-H gel on the GF surface formed a loose network with weaker connections between the gels and more pores. As depicted in Figure 11b,d, when the gel covering the GF fell off, the morphologies of the GF surface before and after the freeze-thaw cycles, including the size of the pits and the gel particles on the pits, were not significantly different. As exhibited in Figure 11e,f, in addition to the C-S-H gel, the cement matrix wrapped with GF also included a small amount of crisscrossed ettringite (an aluminate ferrite trisulphate hydrate phase, Aft). After the freeze-thaw cycles, the surface cement matrix of the GF was not significantly damaged (completely or partially falling off), and the wrapping of GF was good.

Based on the preceding analysis, the freeze-thaw cycles did not cause significant damage to the GF surface, and the deterioration (completely or partially falling off) of the gel layer of cement hydration products surrounding the GF was related to the thickness and density of the gel layer; the denser the gel structure and the thicker the structural layer, the less likely it was to fall off or be damaged after the freeze-thaw cycles.

**Figure 12:**
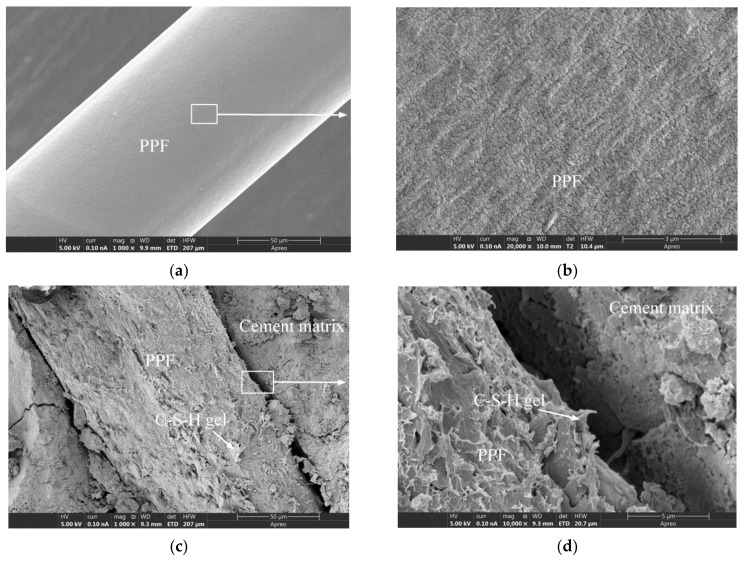
The SEM images of PPF and PPFRC specimens: (**a**) The unused PPF; (**b**) The unused PPF; (**c**) PPF-35-90; (**d**) PPF-35-90.

**Figure 13:**
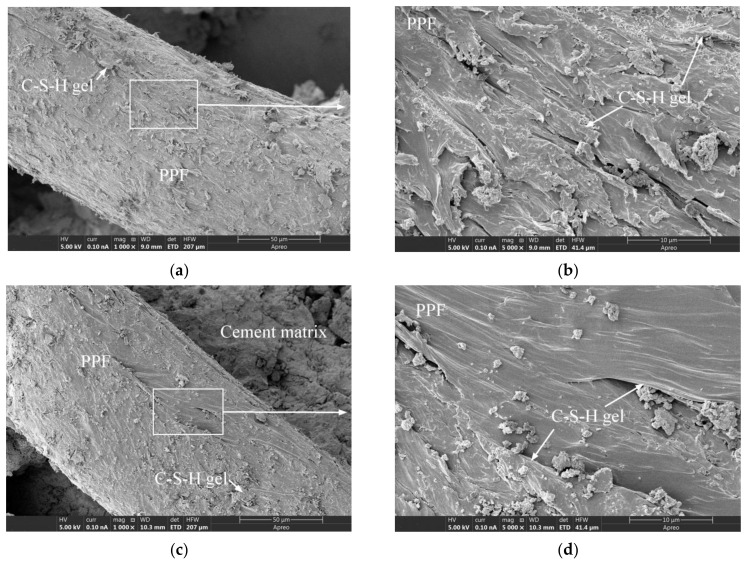
The SEM images of PPFRC specimens before and after freeze-thaw cycles: (**a**) PPF-30-135 before freeze-thaw cycles; (**b**) PPF-30-135 before freeze-thaw cycles; (**c**) PPF-35-135 after freeze-thaw cycles; (**d**) PPF-35-135 after freeze-thaw cycles.

Figure 12a,b depict the micromorphology of unused PPF, from which it is evident that the PPF surface appeared to be smooth at 1000× magnification, but was found to become significantly rougher compared with that of the GF at 20,000× magnification. Figure 12d is a partial enlarged view of Figure 12c. The cement matrix in the interfacial transition zone was smooth and complete, and the gel morphology and structure were significantly different from those of the hydration products bonded to the PPF surface, thereby indicating a significant difference from the GF interfacial transition zone (Figure 10d). Moreover, by comparing Figure 8b,c, Figure 10c,d and Figure 12c,d, it can be seen that the bonding between the PPF and the cement matrix was weaker than that of the GF. A similar conclusion can be drawn by the comparative analysis of reference [14,31,51,52]. PPF does not participate in the cement hydration reaction, as it is chemically inert and very stable in the alkaline environment of concrete [49], indicating that PPFRC is suitable for alkaline environments [1]. Figure 13 presents the microstructures of PPFRC before and after freeze-thaw cycles. After 150 freeze-thaw cycles, the gel structure on the PPF surface exhibited no obvious difference, which also indicates the good frost resistance of PPF.

## 4. Conclusions

This study systematically investigated the mechanical properties, water absorption properties, and fiber degradation of GFRC and PPFRC after natural aging for three years. The following conclusions were reached.

1. The water/binder ratio (w/b) was found to affect the rate of increase of the long-term compressive strength of naturally aged concrete. With the increase of w/b from 0.30 to 0.35, the I_3y-28d_ values of plain concrete, GFRC, and PPFRC with different fiber contents all decreased, and the range of decrease was between 6.58% and 51.96%. In other words, after the increase of w/b, as the contents of GF and PPF increased, the compressive strength of concrete increased more slowly.
2. The type and content of fiber were found to affect the rate of increase of the long-term compressive strength of naturally aged concrete, and the I_3y-28d_ value of the FRC was generally lower than that of plain concrete. The I_3y-28d_ value of the GFRC was lower than that of the PPFRC with the same fiber content. Excluding that of specimen PPF-30-135, the I_3y-28d_ values of the GFRC and PPFRC were lower than those of plain concrete.
3. Compared with the water absorption of the specimens cured for 28 days, that of the FRC specimens cured for three years was significantly reduced, with a range of decrease between 3% and 52.4%. This is due to the subsequent hydration reaction that compacted the internal structure of the concrete. However, with the increases of w/b and the fiber content, the water absorption of specimens GF-35-135 and PPF-35-135 cured for three years increased by 9.1% and 10.9%, respectively, as compared to those of the specimens cured for 28 days. This was due to the large internal pore size and numbers of pores in these two groups of concrete at the initial stage (28 days), and the products produced by the hydration reaction in the later stage (after 28 days) were not sufficient to effectively improve the internal pore structure.
4. With the increase of the curing age, the optimal GF content to reduce the water absorption of concrete was found to decrease. Considering the effect of GF on the water absorption of concrete, 1.35% (w/b = 0.30) and 0.90% (w/b = 0.35) were found to be the optimal GF contents if the water absorption of the specimens cured for 28 days is used to measure the GF effect; however, if the water absorption of the specimens cured for three years is considered, the optimum GF contents were found to be reduced to 0.90% (w/b = 0.30) and 0.45% (w/b = 0.35), respectively.
5. When the natural curing age was three years, the interfacial transition zone between the GF and cement matrix consisted of a small amount of Ca(OH)_2_ and a large amount of flocculent or granular C-S-H gel. The GF surface was degraded into continuous pits with diameters of about 200 to 600 nm, and the surfaces of the pits were attached with spherical granular C-S-H gel products with diameters of about 30 to 44 nm. The cement hydration products in the interfacial transition zone and the products attached to the GF surface were highly consistent in both morphology and size.
6. The freeze-thaw cycles were found to have no significant effect on the pits on the GF surface and the granular C-S-H gel products attached to the pits, but caused a portion of the cement matrix covering the GF to fall off. The factors affecting the shedding area and form of the coating mainly included the compactness and thickness of the coating.
7. SEM observation revealed that the interfacial bonding between the PPF and the cement matrix in the PPFRC exhibited almost no change after curing for three years as compared with that after curing for 28 days. Moreover, the cement hydration products on the PPF surface were not significantly damaged by 150 freeze-thaw cycles.

Compared with the diameter of GF, the degradation pits on the GF surface were relatively shallow after curing for three years, from which it can be inferred that the degree of adverse effects on the compressive strength, water absorption, and durability of concrete was lower. However, it is unclear whether the depth of the degradation pits will further increase with the increase of the curing age. If so, the degrees of influence on the deterioration of the strength and durability of concrete require further study.

## Figures and Tables

**Table 1:** Physical and mechanical properties of cement.

Cement	Soundness	Specific Surface Area (m^2^/kg)	Setting Time (min)		Compressive Strength (MPa)		Flexural Strength (MPa)	
			Initial	Final	3 Days	28 Days	3 Days	28 Days
Experimental result	Qualified	324	159	223	23.6	48.9	6.3	8.1

**Table 2:** Chemical compositions of cement.

Chemical Compositions (%)	SiO_2_	CaO	Al_2_O_3_	Fe_2_O_3_	MgO	SO_3_	Na_2_O	K_2_O	Loss on Ignition
Cement	21.5	59.81	5.86	2.85	2.23	2.06	0.2	0.67	4.82

**Table 3:** Chemical compositions of fly ash.

Chemical Compositions (%)	SiO_2_	CaO	Al_2_O_3_	Fe_2_O_3_	MgO	SO_3_	Na_2_O	K_2_O	Loss on Ignition
Fly ash	66.67	3.05	18.97	4.39	1.24	0.3	-	-	5.38

**Table 4:** Chemical compositions of silica fume.

Chemical Compositions (%)	SiO_2_	CaO	Al_2_O_3_	Fe_2_O_3_	MgO	SO_3_	Na_2_O	K_2_O	Loss on Ignition
Silica fume	93.82	0.41	0.21	0	0.65	0.64	0.32	0.85	3.1

**Table 5:** Chemical compositions of slag.

Chemical Compositions (%)	SiO_2_	CaO	Al_2_O_3_	Fe_2_O_3_	MgO	SO_3_	Na_2_O	K_2_O	Loss on Ignition
Slag	32.08	38.09	15.06	0.94	8.26	0.17	-	-	5.4

**Table 6:** Properties of GF and PPF.

Type of Fiber	Length (mm)	Diameter (μm)	Aspect Ratio	Specific Gravity	Tensile Strength (MPa)	Elastic Modulus (MPa)
GF	12	15	800	2.36	1300	4286
PPF	12	60	200	0.91	486	4800

**Table 7:** The mixture proportions of the concrete mixes.

Mix ID	w/b	Cement (kg/m^3^)	Fly Ash (kg/m^3^)	Silica Fume (kg/m^3^)	Slag Powder (kg/m^3^)	Fine Aggregate (kg/m^3^)	Coarse Aggregate (kg/m^3^)	Water (kg/m^3^)	SP (kg/m^3^)	Volume Fraction of Fiber (%)	
										GF	PPF
PC-30	0.30	280	86	22	43	655	1165	129	6.45	-	-
GF-30-45	0.30	280	86	22	43	655	1165	129	6.45	0.45	-
GF-30-90	0.30	280	86	22	43	655	1165	129	6.45	0.90	-
GF-30-135	0.30	280	86	22	43	655	1165	129	6.45	1.35	-
PPF-30-45	0.30	280	86	22	43	655	1165	129	6.45	-	0.45
PPF-30-90	0.30	280	86	22	43	655	1165	129	6.45	-	0.90
PPF-30-135	0.30	280	86	22	43	655	1165	129	6.45	-	1.35
PC-35	0.35	240	74	18	37	677	1204	129	5.55	-	-
GF-35-45	0.35	240	74	18	37	677	1204	129	5.55	0.45	-
GF-35-90	0.35	240	74	18	37	677	1204	129	5.55	0.90	-
GF-35-135	0.35	240	74	18	37	677	1204	129	5.55	1.35	-
PPF-35-45	0.35	240	74	18	37	677	1204	129	5.55	-	0.45
PPF-35-90	0.35	240	74	18	37	677	1204	129	5.55	-	0.90
PPF-35-135	0.35	240	74	18	37	677	1204	129	5.55	-	1.35

Note: w/b: water/binder ratio, SP: Superplasticizer.

**Table 8:** The rate of increase of the three-year compressive strength of concrete relative to the 28-day compressive strength.

Mix ID	PC-30	GF-30-45	GF-30-90	GF-30-135	PPF-30-45	PPF-30-90	PPF-30-135
I_3y-28d_	89.20%	45.63%	30.09%	49.89%	78.69%	77.42%	96.67%
**Mix ID**	**PC-35**	**GF-35-45**	**GF-35-90**	**GF-35-135**	**PPF-35-45**	**PPF-35-90**	**PPF-35-135**
I_3y-28d_	69.54%	39.05%	4.69%	19.28%	63.80%	56.70%	44.71%

**Table 9:** The water absorption test results of the GFRC and PPFRC cured for three years.

MIX ID	Water Absorption (%)							
	1 Day	2 Days	3 Days	4 Days	7 Days	28 Days	31 Days	37 Days
PC-30	1.65 (−45.0%)	1.78 (−44.5%)	1.86 (−43.8%)	1.92 (−43.2%)	1.96 (−43%)	2.05 (−41.6%)	2.05 (−41.6%)	2.05 (−41.8%)
GF-30-45	1.33 (−24.0%)	1.47 (−27.2%)	1.56 (−28.1%)	1.63 (−27.6%)	1.75 (−25.2%)	2.00 (−20.6%)	2.01 (−19.9%)	2.02 (−20.2%)
GF-30-90	0.98 (−35.9%)	1.07 (−41.2%)	1.14 (−42.4%)	1.17 (−43.5%)	1.26 (−41.9%)	1.53 (−35.2%)	1.54 (−35.0%)	1.54 (−35.6%)
GF-30-135	1.09 (−9.9%)	1.21 (−13.6%)	1.34 (−12.4%)	1.41 (−10.8%)	1.54 (−11%)	1.91 (−3.5%)	1.93 (−3.0%)	1.96 (−2.5%)
PPF-30-45	2.15 (−13.0%)	2.31 (−16.6%)	2.46 (−16.3%)	2.52 (−16.3%)	2.58 (−16.8%)	2.77 (−11.5%)	2.77 (−11.2%)	2.77 (−10.9%)
PPF-30-90	2.44 (−16.4%)	2.67 (−20.8%)	2.84 (−20.2%)	2.93 (−20.4%)	3.00 (−21.3%)	3.18 (−20.5%)	3.17 (−20.6%)	3.17 (−20.2%)
PPF-30-135	3.87 (−9.2%)	4.06 (−16.1%)	4.26 (−16.6%)	4.43 (−14.5%)	4.45 (−16.5%)	4.66 (−15.0%)	4.66 (−14.5%)	4.66 (−13.9%)
PC-35	1.80 (−24.1%)	1.97 (−26.8%)	2.08 (−26.0%)	2.16 (−24.7%)	2.22 (−25.8%)	2.35 (−24.4%)	2.34 (−25.0%)	2.35 (−24.4%)
GF-35-45	1.08 (−48.3%)	1.15 (−52.7%)	1.21 (−53.5%)	1.23 (−54.1%)	1.29 (−54.9%)	1.45 (−52.8%)	1.46 (−52.4%)	1.46 (−52.9%)
GF-35-90	1.30 (−16.1%)	1.39 (−19.7%)	1.49 (−18.6%)	1.56 (−16.6%)	1.67 (−15.7%)	1.98 (−10.8%)	1.99 (−11.6%)	1.99 (−12.7%)
GF-35-135	1.90 (21.8%)	2.03 (8.0%)	2.20 (6.3%)	2.30 (6.5%)	2.55 (5.4%)	3.41 (10.0%)	3.46 (9.1%)	3.50 (8.0%)
PPF-35-45	2.42 (−26.4%)	2.65 (−32.6%)	2.77 (−35.9%)	2.88 (−35.1%)	3.04 (−34.2%)	3.22 (−31.8%)	3.23 (−31.6%)	3.23 (−31.4%)
PPF-35-90	2.31 (−4.9%)	2.53 (−9.3%)	2.69 (−9.1%)	2.81 (−9.1%)	3.12 (−7.4%)	3.72 (−4.4%)	3.73 (−4.4%)	3.72 (−5.6%)
PPF-35-135	3.59 (15.1%)	3.93 (5.9%)	4.24 (6.8%)	4.49 (9.2%)	5.38 (10.2%)	7.06 (12.2%)	7.12 (10.9%)	7.14 (9.5%)

Note: The percentages in parentheses represent the rate of increase of water absorption as compared to the specimens cured for 28 days.

## Data Availability

Not applicable.
